# Growth Medium-Dependent Glycine Incorporation into the Peptidoglycan of *Caulobacter crescentus*

**DOI:** 10.1371/journal.pone.0057579

**Published:** 2013-02-28

**Authors:** Constantin N. Takacs, Jason Hocking, Matthew T. Cabeen, Nhat Khai Bui, Sebastian Poggio, Waldemar Vollmer, Christine Jacobs-Wagner

**Affiliations:** 1 Department of Molecular, Cellular and Developmental Biology, Yale University, New Haven, Connecticut, United States of America; 2 Howard Hughes Medical Institute, Yale University, New Haven, Connecticut, United States of America; 3 Centre for Bacterial Cell Biology, Institute for Cell and Molecular Biosciences, Newcastle University, Newcastle upon Tyne, United Kingdom; 4 Department of Microbial Pathogenesis, Yale School of Medicine, New Haven, Connecticut, United States of America; Centre National de la Recherche Scientifique, Aix-Marseille Université, France

## Abstract

The peptidoglycan (PG) is a macromolecular component of the bacterial cell wall that maintains the shape and integrity of the cell. The PG of *Caulobacter crescentus*, unlike that of many other Gram-negative bacteria, has repeatedly been shown to contain significant amounts of glycine. This compositional peculiarity has been deemed an intrinsic characteristic of this species. By performing a comprehensive qualitative and quantitative analysis of the *C. crescentus* PG by high-performance liquid chromatography (HPLC) and mass spectrometry (MS), we show here that glycine incorporation into the *C. crescentus* PG depends on the presence of exogenous glycine in the growth medium. High levels of glycine were detected at the fifth position of the peptide side chains of PG isolated from *C. crescentus* cells grown in the complex laboratory medium PYE or in defined medium (M2G) supplemented with casamino acids or glycine alone. In contrast, glycine incorporation was undetectable when cells were grown in M2G medium lacking glycine. Remarkably, glycine incorporation into *C. crescentus* peptidoglycan occurred even in the presence of low millimolar to sub-millimolar concentrations of free glycine. High glycine content in the PG had no obvious effects on growth rates, mode of PG incorporation or cell morphology. Hence, the *C. crescentus* PG is able to retain its physiological functions in cell growth and morphogenesis despite significant alterations in its composition, in what we deem to be unprecedented plasticity.

## Introduction

The peptidoglycan (PG) sacculus, which is a mesh-like macromolecule situated on the exterior side of the cytoplasmic membrane, is an essential cellular component of a large variety of bacterial species. It is composed of glycan chains that are connected to each other through crosslinks formed between short peptide side chains [1], [2]. Among its many important functions, the PG is responsible for the maintenance of cellular integrity and for the conservation of species-specific cellular shapes during growth and division [3]. Cellular growth and morphogenesis are tightly connected to the regulated processes of PG synthesis, remodeling and degradation [1], [2], [4], [5], [6]. As a result, genetic or chemical inactivation of PG synthesizing or modifying enzymes (or of regulatory factors of these enzymes) can result in growth inhibition and cell lysis. It is therefore not surprising that many PG inhibitors (e.g., β-lactam antibiotics and others) have become a widely used class of antibiotics in medicine. The innate immune system has also evolved to use PG features as triggers of pathogen recognition pathways and immune cell activation [7], reflecting the near-ubiquitous presence of the peptidoglycan throughout the bacterial kingdom.

Several aspects of PG structure and composition are conserved, though species-specific differences exist [2]. PG synthesis involves an intracellular cascade of enzymatic steps, in which a lipid-linked disaccharide-pentapeptide PG precursor (also known as lipid II) is synthesized [8], [9]. Lipid II is composed of a disaccharide of N-acetylglucosamine (Glc*N*Ac) and N-acetyl muramic acid (Mur*N*Ac), to which the lipid carrier undecaprenyl pyrophosphate and a pentapeptide with the general sequence l-Ala-d-γ-Glu-m-Dap(Lys)-d-Ala-d-Ala (m-Dap, meso-diaminopimelic acid) are attached. Lipid II synthesis initiates with the sequential addition of l-Ala, d-Glu and m-Dap onto UDP-linked Mur*N*Ac. The peptide side chain is completed by the ligation of a d-Ala-d-Ala dipeptide. The resulting Mur*N*Ac-l-Ala-d-γ-Glu-m-Dap-d-Ala-d-Ala moiety is then loaded onto an undecaprenol residue to form lipid I, to which Glc*N*Ac is added to form lipid II. Next, lipid II is flipped across the cytoplasmic membrane to its external side, where it is enzymatically incorporated into the growing PG macromolecule. Transglycosylases polymerize the lipid II disaccharide subunits into PG glycan chains, while transpeptidases crosslink the peptide side chains between adjacent glycan chains. Two major types of transpeptidases have been described. d,d-transpeptidases, which are targets of penicillin and other β-lactam antibiotics, mediate the substitution of the terminal d-Ala of the pentapeptide by the side chain of the m-Dap residue of another peptide, resulting in a d,d- or m-Dap-d-Ala crosslink [10]. l,d-transpeptidases, on the other hand, mediate the substitution of the fourth d-Ala residue of one side chain by the m-Dap residue of a different peptide, resulting in an l,d-, or m-Dap-m-Dap crosslink [11]. Together, these enzymatic activities are involved in major PG biosynthetic processes, which are accompanied by, and likely coordinated with, a wide array of PG lytic processes [1], [4]. Furthermore, numerous proteins without known direct PG synthesizing or hydrolytic activities are also involved in the regulation of PG dynamics [6].

The structural and compositional features of the PG presented above are largely conserved among bacteria [2], [12]. Deviations from this “canonical” model include, but are not limited to: i) enzymatic modifications of the Glc*N*Ac-Mur*N*Ac disaccharide; ii) amino acid substitutions in positions 1, 2 or 3 of the pentapeptide; iii) indirect crosslinkage by interpeptide bridges in some Gram-positive bacteria (i.e. *Staphylococcus aureus*); and iv) various levels of representation of the d,d- and l,d- crosslinks [2], [12], [13]. The composition of the PG, and especially of the peptide side chains, is more conserved among Gram-negative bacteria [2], [14]. In this group of organisms, the side chains are derived from the canonical pentapeptide l-Ala-d-γ-Glu-m-Dap-d-Ala-d-Ala, present on the lipid II PG precursor. During PG growth, several modifications of the pentapeptide are known to occur, including crosslinking of side chains by d,d- and l,d-transpeptidases, removal of the terminal d-Ala by d,d-carboxypeptidases, attachment of Braun’s lipoprotein by l,d-transpeptidases [15], [16], [17], and incorporation of non-canonical d-amino acids (i.e., d-amino acids other than d-Ala or d-Glu) into the PG [18], [19], [20].

The crescent-shaped aquatic Gram-negative α-proteobacterium *Caulobacter crescentus* has been long thought to synthesize the typical Gram-negative PG but with some significant differences. For instance, its PG shows no evidence of a covalent link to Braun’s lipoprotein, and it contains high amounts of pentapeptide [21], [22]. These findings are consistent with the absence of a gene encoding Braun’s lipoprotein from the *C. crescentus* genome, and with the presence of only one d,d-carboxypeptidase-encoding gene [22]. However, the most striking, unexplained feature of the *C. crescentus* PG is the high amount of glycine replacing the terminal d-Ala of the pentapeptide, resulting in the modified peptide side chain l-Ala-d-γ-Glu-m-Dap-d-Ala-Gly or Penta(Gly) instead of the canonical pentapeptide l-Ala-d-γ-Glu-m-Dap-d-Ala-d-Ala, or Penta(Ala) [21], [22]. Penta(Gly) represents roughly half of all pentapeptide side chains observed in the PG of *C. crescentus* [21], [22]. The absolute levels of Penta(Gly) side chains in the PG of *C. crescentus* range from 11–15% [22], [23].

In this study, we perform a comprehensive study of the composition of PG material isolated from *C. crescentus* cells grown in different media. This comparative study demonstrates that glycine incorporation into the *C. crescentus* PG is not an intrinsic property, but is instead the result of the ability of *C. crescentus* to incorporate glycine from the environment even when it is present at low millimolar to sub-millimolar concentrations.

## Methods

### Growth Conditions

Cultures of the *C. crescentus* strain CB15N, also known as NA1000 [24], were grown at 30°C with shaking in the complex medium PYE (0.2% w/v bacto peptone, 0.1% w/v yeast extract, 1 mM MgSO_4_, 0.5 mM CaCl_2_) or in the defined medium M2G (1× M2 salts, 0.5 mM MgSO_4_, 0.5 mM CaCl_2_, 10 µM FeSO_4_, 10 µM EDTA, 0.2% w/v glucose), as previously described [25]. When needed, M2G medium was supplemented with casamino acids (0.1% w/v final concentration) or glycine (0.2 or 2 mM final concentration). Growth curves were generated using a Biotek Synergy2 96 well plate reader. Optical density measurements (at 660 nm) were taken every 2 minutes. Doubling times for each condition were determined by fitting an exponential curve to the data within the largest interval representing exponential growth. Statistics for each condition were calculated from four biological replicates, each grown in duplicate.

### Light Microscopy and Cell Dimensions Analysis

CB15N cultures were grown overnight in the indicated medium, harvested at an OD below 0.3, and spotted on a 1% agarose pad containing the indicated medium. Imaging was performed on a Nikon Eclipse Ti-U equipped with a Hamamatsu Orca-ER LCD camera. Cell outlines, cell lengths and widths were determined using Matlab and the open source image analysis software MicrobeTracker [26].

### Muropeptide Analysis

*C. crescentus* muropeptides were obtained as previously described [15], [22]. Briefly, one-liter cultures of CB15N were grown in the indicated media to OD_660_ values between 0.3 and 0.4, cooled on ice for 10 min, pelleted by centrifugation at 10,400×g and 4°C for 10 min, resuspended in cold M2 salts, and added drop-wise to boiling 8% SDS solution. The resulting suspension was further boiled for 30 min with vigorous stirring while water was added to maintain a relatively constant volume. The resulting peptidoglycan sacculi were pelleted at 440,000×g for 15 min in a Beckman Coulter Optima TLX ultracentrifuge and repeatedly washed with water until free of SDS, as assayed using the Hayashi test [27].

The PG sacculi were next incubated with 100 µg/ml amylase in 10 mM Tris-HCl buffer (pH 7.0) for 2 h at 37°C, and then with 200 µg/ml pronase for 1 h at 60°C (pronase was pre-incubated for 2 h at 60°C). The enzymes were removed by boiling for 30 min in 4% SDS, and the sacculi were washed free of SDS as above and stored at 4°C with 0.02% sodium azide in water. Muropeptides were then obtained by digestion of the sacculi with 20 µg/ml cellosyl in 20 mM sodium phosphate, pH 4.8, overnight at 37°C. Cellosyl was removed by heating the reaction mixture at 100°C for 10 min, and centrifuging (15,000×g for 30 min). The muropeptides were reduced with sodium borohydride in 0.25 M sodium borate, pH 9.0, for 30 min, and the pH of the samples was adjusted between 3.0 and 4.5 using 20% phosphoric acid. The samples were then concentrated under vacuum at RT in a Rotovap, and analyzed by HPLC on an Agilent 1200 system equipped with a 250 mm by 4.6 mm, 3 µm C_18_ Prontosil ODS column maintained at 55°C. The solvent system used was a 135 min linear gradient from solvent A (50 mM sodium phosphate, pH 4.31), to solvent B (made by mixing 75 mM sodium phosphate buffer, pH 4.95 and methanol in a 7∶3 volume ratio). UV absorbance was quantified at 205 nm. Major muropeptide peak fractions were manually collected and analyzed by tandem mass spectrometry (MS/MS) as previously described [28]. Some muropeptides were identified based on their previously determined retention times and their order of elution [22]. All calculations were performed as previously described [15], [22]. Specifically, for each run, the areas corresponding to all known peaks were reported as percentages of their sum. Next, these percentage values were adjusted for different UV absorbance properties of the various muropeptide species by multiplication with empirically calculated extinction coefficients [15], and re-normalized with respect to their sum. The abundance of each individual class of PG features was also calculated using previously described formulae [15].

### d-cysteine Pulse-chase Experiments

One-liter cultures of CB15N were diluted to an OD_660_ of ∼0.001 in M2G medium and allowed to grow to OD_660_ ∼0.03. At this time, d-cysteine (d-Cys) was added to the medium at a final concentration of 125 µg/ml, and the culture was allowed to grow until the OD_660_ reached 0.56 (approximately 21 h). The cells were then collected by centrifugation and split into two samples; one was used to inoculate a fresh 1 L M2G culture for the chase and the other was processed immediately. Chase samples were collected 90, 150, or 210 min after the labeling (OD_660_ values at harvest were 0.14–0.26). Purification and biotinylation of the sacculi were performed as described [29], [30]. Immunolabeling, signal amplification, and electron microscopy were performed as described [31].

## Results

### Glycine Incorporation into *C. crescentus* Peptidoglycan is Growth Medium-dependent

A widely used method to determine the composition and infer the structure of bacterial PG relies on separation of the sacculi from other cellular components such as nucleic acids, proteins and lipids, followed by digestion of the glycan chains with the enzyme muramidase [15]. Next, the resulting PG fragments, also known as muropeptides, are reduced, chromatographically separated and quantified. Their composition and structure can then be analyzed by chemical or mass spectrometry (MS) techniques [15], [28]. Importantly, this sample preparation procedure leaves intact the peptide side chains found in the analyzed PG, as well as the crosslinks connecting them. Using these techniques, it has been shown that *C. crescentus* PG samples contain high levels of pentapeptide side chains and that roughly half of them terminated with a glycine residue [21]. It has therefore been assumed since then that the PG of *C. crescentus* is characterized by a high Penta(Gly) peptide content, an idea supported by later PG analyses [22], [31], [32].

**Figure 1:**
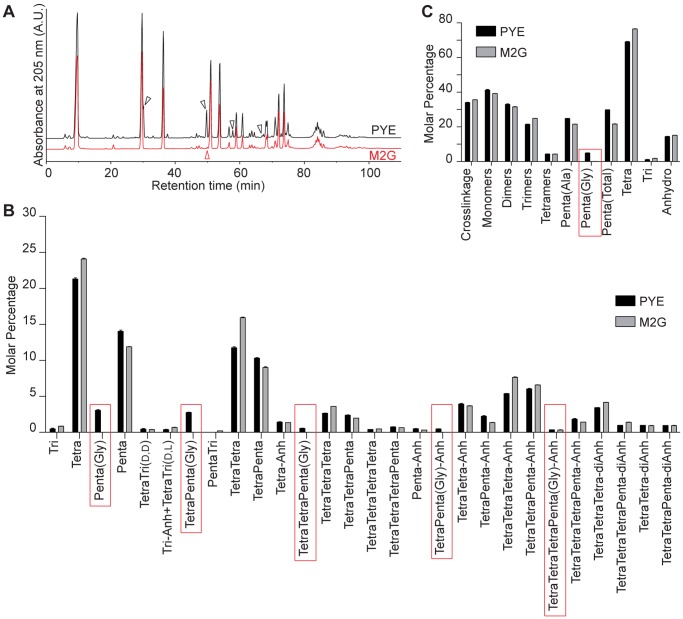
Comparison between the PG composition of *C. crescentus* cells grown in PYE and M2G media. (A). Overlay of HPLC profiles of muropeptides obtained from *C. crescentus* cultures grown in PYE (black trace) and M2G (red trace) media. Black arrowheads, Penta(Gly)-containing muropeptide peaks present only in the PYE-derived sample; red arrowhead, peak corresponding to the PentaTri muropeptide species, identified in the M2G-derived sample. (B). Relative representation (molar percentage) of each muropeptide species in PG digests obtained from *C. crescentus* cultures grown in PYE (black) and M2G (grey) media. Red rectangles denote glycine-containing species. Tri, Glc*N*Ac-Mur*N*Ac-l-Ala-d-γ-Glu-m-Dap; Tetra, Glc*N*Ac-Mur*N*Ac-l-Ala-d-γ-Glu-m-Dap-d-Ala; Penta, Glc*N*Ac-Mur*N*Ac-l-Ala-d-γ-Glu-m-Dap-d-Ala-d-Ala; Penta(Gly), Glc*N*Ac-Mur*N*Ac-l-Ala-d-γ-Glu-m-Dap-d-Ala-Gly; Anh, 1,6-anhydro- Mur*N*Ac; (D,D), m-Dap-D-Ala crosslink; (L,D), m-Dap-m-Dap crosslink. Bars represent averages ± standard deviation for the three samples analyzed. (C). Summary of the composition of *C. crescentus* PG digests obtained from cultures grown in the indicated media. Major PG characteristics are shown, namely the total degree of crosslinkage, the relative amounts of differentially crosslinked muropeptide classes (monomers, dimers, trimers and tetramers), side chain types (Tri, l-Ala-d-γ-Glu-m-Dap; Tetra, l-Ala-d-γ-Glu-m-Dap-d-Ala; Penta(Ala), l-Ala-d-γ-Glu-m-Dap-d-Ala-d-Ala; Penta(Gly), l-Ala-d-γ-Glu-m-Dap-d-Ala-Gly), or chain ends (anhydro, 1,6-anhydro-Mur*N*Ac ). Bars are as in (B). The red rectangle highlights the relative representation of the Penta(Gly) side chain. (B and C).

However, all published muropeptide analyses have been conducted on PG isolated from cultures grown in the complex medium PYE. After performing a control experiment for a cell wall hydrolase mutant strain [33] that can grow in the defined medium M2G but not in PYE, we became suspicious that the PG composition may be sensitive to the composition of the growth medium (data not shown). Therefore, we performed a thorough comparative analysis of PG from wild-type CB15N cultures grown in either PYE or M2G (n = 3 for each). As expected, we identified both free and crosslinked forms of Penta(Gly)-containing muropeptides in the samples we obtained from PYE-grown cultures (Fig. 1A, black trace and arrowheads, Fig. 1B, red rectangles, and Table 1). In contrast, these glycine-containing muropeptides were virtually undetectable in samples obtained from M2G-grown cultures (Fig. 1A, red trace, Fig. 1B, and Table 1). Penta(Gly) peptides represented 4.96±0.13% of all side chains and 17% of all pentapeptides within the PG of PYE-grown cells (Fig. 1C and Table 2). Their presence represented the major difference between the muropeptide profiles of *C. crescentus* PG obtained from PYE and M2G cultures.

**Table 1:** Quantification of individual muropeptides identified in the PG of *C. crescentus* cells grown in PYE or M2G media.

Muropeptide	Retention Time (min)	Molar Percentage (Mean ± Standard deviation)	
		PYE (n = 3)	M2G(n = 3)
Tri	20.7	0.48±0.19	0.87±0.04
Tetra	29.6	21.32±0.25	24.08±0.20
Penta(Gly)	30.1	3.05±0.19	N.D.*
Penta	36.2	14.05±0.27	11.88±0.03
TetraTri(D,D)	46.5	0.46±0.13	0.39±0.03
Tri-Anh (+ small amount of TetraTri(D,L))	47.3	0.38±0.08	0.69±0.02
TetraPenta(Gly)	49.7	2.76±0.08	N.D.*
PentaTri(D,L)	49.8	N.D.*	0.20±0.02
TetraTetra	50.9	11.77±0.19	15.95±0.10
TetraPenta	53.7	10.31±0.11	9.02±0.18
Tetra-Anh	56.6	1.44±0.06	1.35±0.04
TetraTetraPenta(Gly)	57.7	0.57±0.02	N.D.*
TetraTetraTetra	58.8	2.67±0.05	3.62±0.01
TetraTetraPenta	60.7	2.38±0.11	1.98±0.03
TetraTetraTetraTetra	63.0	0.42±0.01	0.49±0.01
TetraTetraTetraPenta	63.6	0.76±0.07	0.66±0.01
Penta-Anh	64.4	0.52±0.05	0.34±0.02
TetraPenta(Gly)-Anh	66.8 & 67.3	0.48±0.04	N.D.*
TetraTetra-Anh	68.0 & 68.4	3.94±0.18	3.69±0.03
TetraPenta-Anh	70.9 & 71.1	2.25±0.16	1.37±0.03
TetraTetraTetra-Anh	72.0	5.38±0.08	7.64±0.13
TetraTetraPenta-Anh	73.7	6.04±0.15	6.58±0.07
TetraTetraTetraPenta(Gly)-Anh	74.2	0.36±0.02	0.34±0.01
TetraTetraTetraPenta-Anh	74.9	1.83±0.20	1.43±0.00
TetraTetraTetra-diAnh	83.5 & 84.0	3.45±0.02	4.15±0.05
TetraTetraTetraPenta-diAnh	84.4	0.99±0.01	1.40±0.05
TetraTetra-diAnh	85.0	0.99±0.01	0.95±0.04
TetraTetraPenta-diAnh	85.8	0.95±0.00	0.94±0.07

Nomenclature of muropeptides as established by Glauner [15]. *N.D. Peak not detected.

**Table 2:** Summarized composition of the PG of *C. crescentus* cells grown in PYE or M2G media.

Peptidoglycan feature	Molar percentage (mean ± standard deviation)	
	PYE (n = 3)	M2G (n = 3)
Monomers	41.25±0.71	39.21±0.14
Dimers	32.97±0.55	31.56±0.22
Trimers	21.43±0.34	24.91±0.16
Tetramers	4.35±0.14	4.32±0.07
Cross-linkage	34.04±0.37	35.63±0.07
Pentapeptides (Ala5)	24.86±0.23	21.55±0.17
Pentapeptides (Gly5)	4.96±0.13	0.09±0.00
Pentapeptides (Total)	29.82±0.18	21.64±0.17
Tetrapeptides	69.09±0.38	76.51±0.16
Tripeptides	1.09±0.21	1.85±0.05
Anhydro	14.45±0.16	15.14±0.13
	Number of disaccharide subunits (mean ± standard deviation)	
	PYE (n = 3)	M2G (n = 3)
Average glycan chain length	6.92±0.08	6.61±0.06

There were other discernible differences. For example, the relatively abundant TetraPenta(Gly) dimer present among the muropeptides isolated from PYE-grown cells (retention time 49.7 min, Fig. 1A and Table 1) was replaced in the M2G-grown sample by a significantly smaller peak with a similar retention time (49.9 min, Fig. 1A, red arrowhead). We identified the muropeptide from this peak as the (l,d)-crosslinked PentaTri dimer, which was likely masked in the chromatogram of the PYE-based sample by the more abundant TetraPenta(Gly) dimer. We also observed overall higher amounts of pentapeptides (∼30% vs. ∼22%) and lower amounts of tetrapeptides (∼69% vs. ∼77%) in the PG of PYE-grown cells compared to that of M2G-grown cells (Fig. 1C and Table 2). Tetrapeptide side chains may form as a result of two enzymatic pathways: either D,D-carboxypeptidase-mediated removal of the terminal D-Ala of the pentapeptide, or transpeptidase-mediated crosslinkage (D,D-transpeptidation) of two side chains followed by cleavage of the crosslink by an endopeptidase. The apparent inversely correlated differences between the levels of these two types of side chains in the PG of PYE- and M2G-grown samples could be explained by the presence of the Penta(Gly) species, if this peptide functions as a less efficient substrate for either the D,D-carboxypeptidase or the transpeptidases of *C. crescentus*. These differences were, however, not associated with major effects on the degree of PG crosslinkage or on the average length of the glycan chains (Fig. 1C and Table 2). This result suggests that the *C. crescentus* PG is tolerant to some changes in its composition.

### Addition of Glycine to M2G Medium Results in its Incorporation into the PG

**Figure 2:**
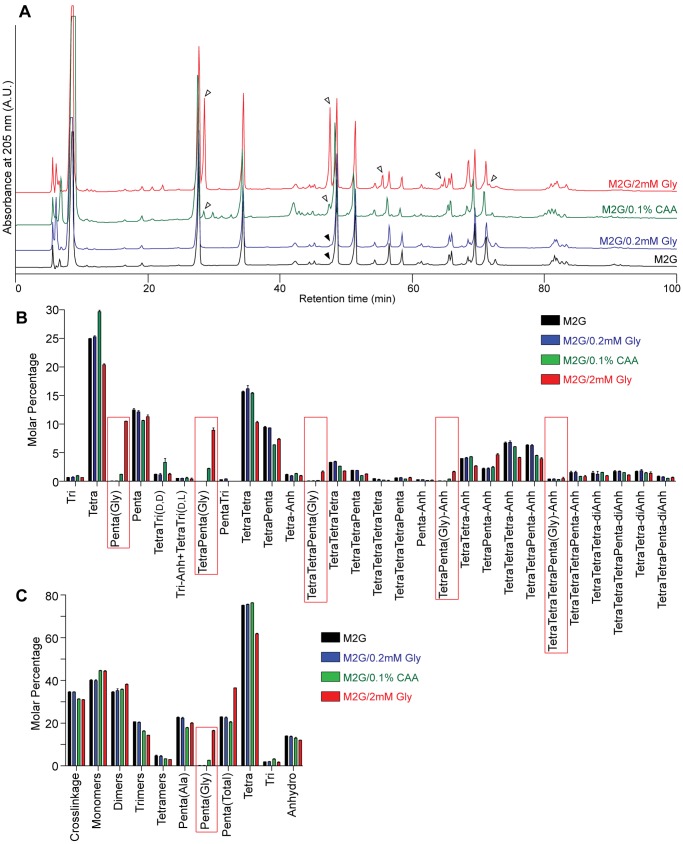
Comparison between the PG composition of *C. crescentus* cells grown in unsupplemented and glycine-supplemented M2G media. (A). Overlay of HPLC profiles of muropeptides obtained from *C. crescentus* cultures grown in the indicated media. CAA, casamino acids; empty arrowheads, Penta(Gly)-containing muropeptide peaks; filled arrowheads, peak corresponding to the PentaTri muropeptide species. (B). Relative representation (molar percentage) of each muropeptide species in PG digests obtained from *C. crescentus* cultures grown in the indicated media. Red rectangles denote glycine-containing species; Tri, Glc*N*Ac-Mur*N*Ac-l-Ala-d-γ-Glu-m-Dap ; Tetra, Glc*N*Ac-Mur*N*Ac-l-Ala-d-γ-Glu-m-Dap-d-Ala; Penta, Glc*N*Ac-Mur*N*Ac-l-Ala-d-γ-Glu-m-Dap-d-Ala-d-Ala; Penta(Gly), Glc*N*Ac-Mur*N*Ac-l-Ala-d-γ-Glu-m-Dap-d-Ala-Gly; Anh, 1,6-anhydro- Mur*N*Ac; (D,D), m-Dap-D-Ala crosslink; (L,D), m-Dap-m-Dap crosslink. Bars represent averages ± standard deviation for two (M2G, M2G + 0.2 mM Gly, M2G + 2 mM Gly) or three (M2G + 0.1% CAA) samples analyzed. (C). Summary of the composition of *C. crescentus* PG digests obtained from *C. crescentus* cultures grown in the indicated media. Major PG characteristics are shown, namely the total degree of crosslinkage, the relative amounts of differentially crosslinked muropeptide classes (monomers, dimers, trimers and tetramers), side chain types (Tri, l-Ala-d-γ-Glu-m-Dap ; Tetra, l-Ala-d-γ-Glu-m-Dap-d-Ala; Penta(Ala), l-Ala-d-γ-Glu-m-Dap-d-Ala-d-Ala; Penta(Gly), l-Ala-d-γ-Glu-m-Dap-d-Ala-Gly), or chain ends (anhydro, 1,6-anhydro-Mur*N*Ac ). The red rectangle highlights the relative amounts of the Penta(Gly) side chain. Bars are as in (B).

Our finding that the Penta(Gly) side chain was present in the PG of *C. crescentus* cells grown in the complex medium PYE but not in the minimal medium M2G suggested that the incorporation of glycine may depend on the presence of this amino acid in the growth medium. Furthermore, three studies of *C. crescentus* PG composition identified different values for the Penta(Gly) levels in the PG of PYE-grown cultures: ∼11%, as calculated from the data reported in a 1983 study [21] and for cultures grown in unsupplemented PYE [22], ∼15%, for cultures grown in PYE supplemented with 20 mM Tris-HCl buffer pH 8.0 [22], and ∼5% for the PYE-grown samples analyzed in this study. As the sources of nutrients in PYE medium are enzymatic digests of animal tissue (peptone) and yeast [25], we expect batch-to-batch variations in the concentrations of the different components of this complex medium, including glycine. Based on an analysis of commercially available peptone and yeast extract [34], we calculated that PYE medium made with Difco nutrients contains approximately 4.6 mM total glycine, including 0.3 mM free glycine. The M2G medium formulation, on the other hand, does not include any amino acids [25]. We thus hypothesized that addition of relatively small amounts of exogenous glycine to the otherwise glycine-free growth medium M2G would cause incorporation of this amino acid into the *C. crescentus* PG. Consistent with this hypothesis, growing cells in M2G medium supplemented with 2 mM glycine was sufficient to generate high levels of glycine incorporation into the PG, as visualized and quantified from HPLC traces (Fig. 2A and 2B, Table 3). The Penta(Gly) side chains represented 16.5±0.3 % of all the side chains and 45% of all pentapeptides in these samples (Fig. 2C and Table 4). Other differences in *C. crescentus* PG composition that were observed following growth in glycine-supplemented M2G medium were small. Namely there were slight decreases in the degree of crosslinkage and in the amounts of trimers, tetramers, and tetrapeptides, while monomer and dimer levels and the average length of the glycan chains were only mildly increased (Fig. 2C and Table 4).

**Table 3:** Changes in the muropeptide composition of *C. crescentus* PG following amino acid supplementation of the defined growth medium M2G.

Muropeptide	Molar Percentage (Mean ± Standard deviation)			
	M2G (n = 2)	M2G + 0.2 mM Gly(n = 2)	M2G + 0.1% CAA(n = 3)	M2G + 2 mM Gly(n = 2)
Tri	0.68±0.02	0.68±0.15	0.97±0.05	0.66±0.01
Tetra	24.97±0.07	25.28±0.28	29.68±0.44	20.39±0.23
Penta(Gly)	0.03±0.04	0.05±0.01	1.20±0.06	10.50±0.05
Penta	12.49±0.31	12.13±0.32	10.61±0.13	11.29±0.44
TetraTri(D,D)	1.20±0.08	1.14±0.33	3.31±1.19	1.24±0.21
Tri-Anh (+ small amount of TetraTri(D,L))	0.51±0.00	0.50±0.06	0.56±0.24	0.38±0.25
TetraPenta(Gly)	N.D.*	N.D.*	2.20±0.13	8.94±0.58
PentaTri(D,L)	0.29±0.01	0.41±0.06	N.D.*	N.D.*
TetraTetra	15.67±0.17	16.20±0.79	15.42±0.20	10.29±0.32
TetraPenta	9.47±0.20	9.33±0.06	6.36±0.10	7.37±0.17
Tetra-Anh	1.16±0.11	0.95±0.08	1.34±0.07	0.97±0.09
TetraTetraPenta(Gly)	0.06±0.02	0.05±0.07	0.16±0.00	1.64±0.24
TetraTetraTetra	3.32±0.04	3.42±0.13	2.63±0.04	1.79±0.06
TetraTetraPenta	1.92±0.04	1.88±0.04	0.98±0.07	1.27±0.04
TetraTetraTetraTetra	0.45±0.03	0.28±0.12	0.22±0.01	0.10±0.14
TetraTetraTetraPenta	0.60±0.01	0.60±0.10	0.40±0.01	0.60±0.14
Penta-Anh	0.28±0.01	0.28±0.01	0.17±0.01	0.19±0.05
TetraPenta(Gly)-Anh	0.08±0.01	0.03±0.04	0.38±0.06	1.67±0.14
TetraTetra-Anh	3.99±0.01	4.07±0.16	4.29±0.10	2.66±0.11
TetraPenta-Anh	2.19±0.18	2.21±0.19	2.43±0.31	4.65±0.27
TetraTetraTetra-Anh	6.71±0.21	6.81±0.36	6.03±0.12	4.13±0.10
TetraTetraPenta-Anh	6.32±0.12	6.26±0.20	4.49±0.13	3.95±0.26
TetraTetraTetraPenta(Gly)-Anh	0.38±0.03	0.39±0.05	0.31±0.05	0.46±0.37
TetraTetraTetraPenta-Anh	1.54±0.31	1.54±0.31	0.83±0.10	0.80±0.30
TetraTetraTetra-diAnh	1.44±0.40	1.24±0.72	1.53±0.07	0.96±0.07
TetraTetraTetraPenta-diAnh	1.72±0.20	1.70±0.15	1.51±0.05	1.08±0.08
TetraTetra-diAnh	1.71±0.10	1.83±0.29	1.47±0.11	1.38±0.37
TetraTetraPenta-diAnh	0.83±0.15	0.73±0.11	0.50±0.05	0.64±0.15

* N.D. Not detected.

**Table 4:** Summary of changes in the composition of *C. crescentus* PG following amino-acid supplementation of the defined growth medium M2G.

Peptidoglycan feature	Molar Percentage (Mean ± Standard deviation)			
	M2G(n = 2)	M2G + 0.2 mM Gly(n = 2)	M2G + 0.1% CAA(n = 3)	M2G + 2 mM Gly(n = 2)
Monomers	40.10±0.34	39.88±0.58	44.54±0.39	44.38±0.33
Dimers	34.59±0.22	35.21±1.30	35.85±0.34	38.19±0.22
Trimers	20.60±0.10	20.39±0.28	16.33±0.31	14.38±0.09
Tetramers	4.70±0.46	4.52±0.44	3.28±0.20	3.04±0.01
Cross-linkage	34.56±0.30	34.59±0.14	31.27±0.28	30.97±0.18
Pentapeptides (Ala5)	22.73±0.22	22.31±0.62	17.85±0.36	20.06±0.25
Pentapeptides (Gly5)	0.18±0.03	0.18±0.07	2.63±0.11	16.47±0.29
Pentapeptides (Total)	22.91±0.19	22.49±0.55	20.47±0.47	36.52±0.04
Tetrapeptides	75.16±0.16	75.56±0.33	76.33±0.18	61.81±0.41
Tripeptides	1.93±0.03	1.95±0.22	3.19±0.32	1.66±0.37
Anhydro	13.99±0.17	13.73±0.34	12.99±0.65	12.02±0.11
	Disaccharide subunits/glycan chain (mean ± standard deviation)			
Glycan chain length	7.15±0.09	7.29±0.18	7.71±0.39	8.32±0.08

We were also able to identify the Penta(Gly) peaks when we grew the cells in M2G medium supplemented with 0.1% casamino acids (CAA), which represent a more complex source of amino acids (Fig. 2A). In this case, the Penta(Gly) levels were low but detectable, consisting of 2.63±0.11% of all side chains and 13% of all pentapeptides (Fig. 2B and 2C, Tables 3 and 4). A CAA concentration of 0.1% is estimated to yield 0.16 mM free glycine [35]. When we supplemented M2G medium with a concentration of glycine (0.2 mM) similar to that in M2G + 0.1% CAA medium, we failed to detect significant amounts of Penta(Gly) muropeptides (Fig. 2A, 2B and 2C; Tables 3 and 4). It is possible that the availability of other amino acids in the CAA solution enhances the incorporation of Gly in the PG (see Discussion). In any case, our results indicate that growth of *C. crescentus* in media containing low concentrations of glycine is sufficient to cause incorporation of this amino acid into the PG in the form of a Penta(Gly) peptide side chain.

Because addition of high levels (0.05% or higher) of glycine to the PYE medium (resulting in ∼11 mM total glycine) have been shown to cause morphological abnormalities of *C. crescentus* cells, growth delays, and even cell lysis [23], [36], we tested whether the low levels (up to 2 mM) of glycine that we used in our experiments also affected the growth rate or morphology of *C. crescentus* cells (Table 5). We found no effect on cell length or width, and growth rates were not substantially affected by addition of 0.2 or 2 mM glycine to M2G medium. Addition of 0.1% casamino acids caused an increase in the growth rate, likely due to the casamino acids acting as an additional nutrient source.

**Table 5:** Cell dimensions and growth rates of *C. crescentus* in various growth media.

Growth medium	Estimated Gly conc. (mM)	Cell width (µm)(Mean ± SD)	Cell length (µm)(Mean ± SD)	Doubling time (min)(Mean ± SD)
PYE	4.6	0.72±0.02	2.88±0.67	104±1
M2G	0	0.70±0.03	2.79±0.64	120±2
M2G + 0.2 mM Gly	0.2	0.71±0.02	2.79±0.64	119±3
M2G + 0.1% CAA	0.16	0.71±0.03	2.71±0.60	113±2
M2G + 2 mM Gly	2	0.71±0.02	2.88±0.71	124±3

### New PG Incorporation Occurs at Midcell during Growth in Defined M2G Medium

**Figure 3:**
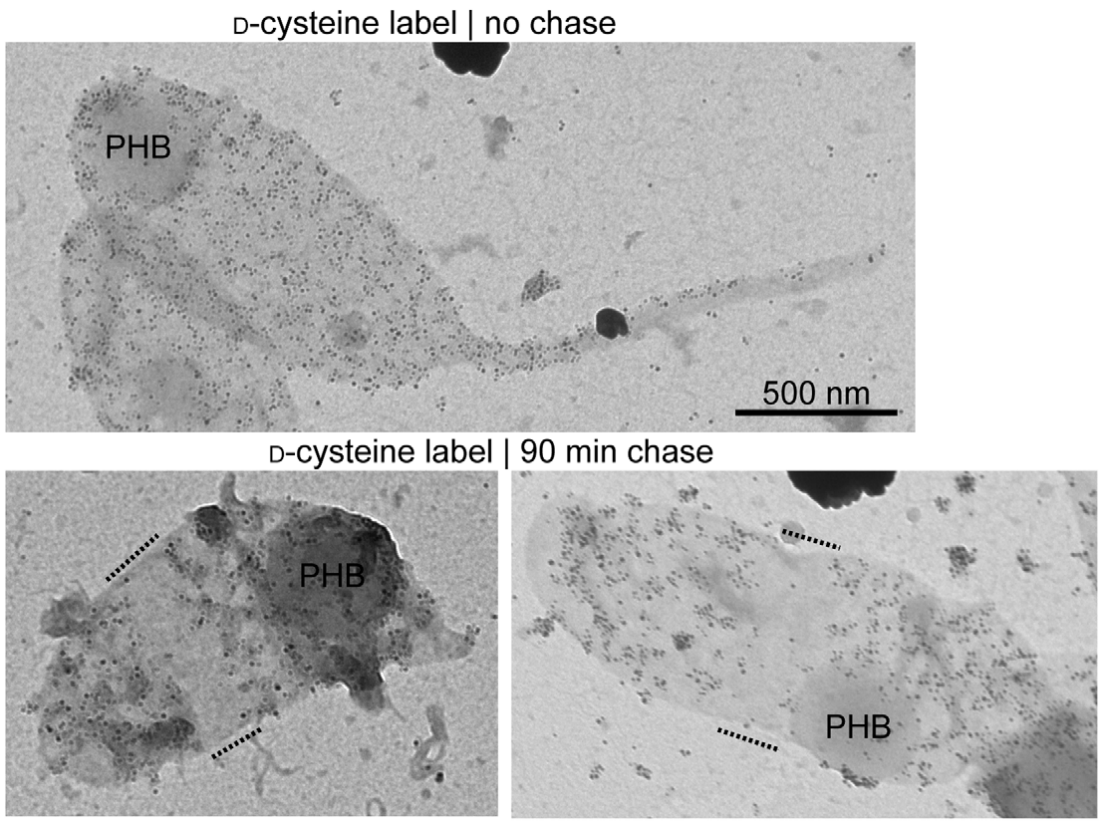
D-cysteine labeling of PG sacculi. Transmission electron micrographs of d-Cys-labeled sacculi either before (top) or after (bottom) a 90-min chase period. These sacculi were obtained from cells grown in M2G medium. d-Cys residues were biotinylated and visualized by silver enhancement of nanogold-coupled anti-rabbit secondary antibody bound to anti-biotin primary antibody. Sacculi were counterstained with uranyl acetate. Dashed lines indicate areas of label clearing. PHB, polyhydroxybutyrate granules.

In *C. crescentus*, FtsZ-dependent preseptal PG growth contributes significantly to cell elongation [29]. This conclusion was in part based on pulse-chase d-cysteine (d-Cys) labeling experiments on cells grown in PYE medium. Given the differences between the composition of PG isolated from cells grown in PYE and M2G media, it became important to test whether preseptal PG growth also occurred in cells grown in M2G media, which do not incorporate Gly into the PG. Therefore, we performed d-Cys pulse-chase labeling experiments in M2G cultures. Repeating these experiments with M2G cultures turned out to be challenging, as the incorporation of d-Cys was considerably less efficient in M2G than in PYE (data not shown). However, we were able to obtain sufficient labeling of PG sacculi of wild-type cells after growth in M2G with 125 µg/ml d-Cys (Fig. 3). By chasing labeled cells for 90 min in the absence of d-Cys, we observed midcell clearing of the d-Cys label in sacculi with little or no midcell constriction (Fig. 3), consistent with preseptal growth occurring in the M2G medium, and similar to what is observed in cells grown in PYE [29]. Thus, preseptal PG elongation is not correlated with glycine incorporation and is a general mode of growth in *C. crescentus.* This notion is supported by the observation that MurG, the enzyme that produces the PG precursor lipid II, is recruited early to the FtsZ ring in non-constricting cells in M2G medium [29], [37].

## Discussion

Early studies [21] suggested that high levels of glycine incorporation into the *C. crescentus* PG (as a Penta(Gly) side peptide) is a general characteristic of this bacterium. Our results indicate that this is unlikely. We show that the PG of *C. crescentus* cells grown in amino acid-free M2G medium contains very low levels of Penta(Gly) side chains, if any. Rather, efficient incorporation of glycine into the *C. crescentus* PG requires its presence in the growth medium at concentrations in the sub-millimolar to low millimolar range, as found in the complex medium PYE or when exogenously added to the amino acid-free medium M2G.

Since the 1983 *C. crescentus* study, Penta(Gly) side chains have been identified in the PG of other species, although we note that in every case, cultures were grown in complex media. From published muropeptide analysis of PG from other species, we calculate Penta(Gly) levels of ∼7% in *S. aureus* [38], 0.4% in *E. coli* [16] and ∼5% [39], [40] or ∼10% [41] in *Helicobacter pylori*. Penta(Gly) side chains were also identified in the PG of *Staphylococcus haemolyticus* [42] and of *Neisseria gonorrhoeae* [43], but their relative abundance was not quantified. Based on our findings, we suspect that the glycine in the PG of these various bacteria is not an intrinsic feature and is medium-specific. In agreement with this idea, supplementing complex media with very high concentrations (50 mM to 1.33 M) of glycine results in Penta(Gly) side chain formation in various bacterial species [38], [44].

How are the Penta(Gly) side chains synthesized? Interestingly, non-canonical d-amino acids produced by a plethora of bacterial species [19], [20] have been shown to replace d-Ala at the fifth position of the pentapeptide [18]. Specifically in the case of *C. crescentus*, growth in PYE medium supplemented with 2 mM D-Met results in its incorporation into the PG as Penta(D-Met) at levels representing ∼10% of all muropeptides [18]. Incorporation of D-amino acids into pentapeptides occurs during an early cytoplasmic step of PG precursor synthesis, specifically during the formation of the d-Ala-d-Ala dipeptide by d-Ala-d-Ala ligases (Ddls) [18]. Non-canonical D-amino acids replace the second d-Ala residue of the dipeptide, and are thereby introduced into the PG precursor pathway [18]. It is likely that glycine incorporation also occurs at this step, resulting in the formation of a D-Ala-Gly peptide which is then utilized by downstream biosynthetic enzymes, resulting in the formation of a modified lipid II PG precursor. Indeed, glycine’s achiral center may formally be viewed as either a “d” form or an “l” form, and thus could serve as a substrate for a promiscuous Ddl. Supporting this hypothesis, several purified Ddls have been shown to be able to use glycine as substrate in *in vitro* assays, albeit with lower affinity than the normal substrate d-Ala [45]. Furthermore, the *E. coli* MurF enzyme, which catalyzes the addition of the d-Ala-d-Ala peptide onto Mur*N*Ac-l-Ala-d-γ-Glu-m-Dap during the cytoplasmic steps of PG precursor synthesis, has been shown to be able to use the D-Ala-Gly peptide as a substrate [46]. Thus, while we cannot exclude other mechanisms, we suggest that, in the presence of sub-millimolar to low millimolar concentrations of free glycine in the growth medium, the *C. crescentus* Ddl synthesizes a mix of d-Ala-d-Ala and d-Ala-Gly peptides, which are then added to Mur*N*Ac-l-Ala-d-γ-Glu-m-Dap by MurF, helping to create a pool of Penta(Gly)-containing PG precursors that are incorporated into the PG in downstream biosynthetic steps.

Our study reveals that in *C. crescentus*, very low (down to sub-millimolar) concentrations of glycine in the medium yield high levels (up to ∼15% of all side chains) of glycine incorporation in the PG; PYE and 0.01% CAA solutions are estimated to contain only 0.3 and 0.16 mM of free glycine. In contrast, the PG of *E. coli* cells grown in LB or PB media, which contain estimated 0.9 mM of free glycine, display Penta(Gly) levels below 0.5% [16]. Similarly, the PG of *Bacillus subtilis* cells grown in LB or nutrient broth (0.9 mM free glycine) has levels of Penta(Gly) either undetectable or below 3%, respectively [47]. The PG of both *E. coli* and *B. subtilis* have low overall levels of pentapeptides because of D,D-carboxypeptidase activity. However, deletion of the *B. subtilis dacA* gene, which encodes a D,D-carboxypeptidase, results in a dramatic (∼40-fold) increase in Penta(Ala) that is accompanied by only a comparatively mild (∼2.7-fold) increase in Penta(Gly) [47]. Thus, *C. crescentus* appears more efficient (or less intolerant) at incorporating glycine into its PG. We note that higher glycine concentrations are needed to drive glycine incorporation into the PG of *C. crescentus* when this amino acid is added alone (M2G + 2 mM glycine) than when it is added as part of a more complex amino acid mixture (PYE or M2G + 0.1% CAA condition). This may reflect competition between glycine incorporation into the PG and its utilization in catabolism and other amino acid synthetic pathways; such competition might be relieved if other amino acids and nutrients are provided in complex media.

*C. crescentus* cells grown in M2G medium must also contain glycine in their cytoplasm as they produce it for protein synthesis; yet they do not incorporate it into the PG. Perhaps the glycine concentration in the cytoplasm of M2G-grown cells is too low to be used by the Ddl enzyme but high enough for the protein synthesis machinery.

The physiological significance of glycine incorporation into *C. crescentus* PG remains elusive. The natural habitats of *Caulobacter* species, mostly aquatic environments [48], [49], are poor in nutrients, and are therefore unlikely to contain concentrations of glycine sufficient for PG incorporation. However, glycine incorporation into the PG robustly occurs during growth in the widely used laboratory medium PYE. We note that very high (10–100 mM) concentrations of glycine in laboratory growth media are detrimental to *C. crescentus*, as they lead to morphological alterations, decreased growth rates, and cell lysis [36]. Similar phenotypic effects have been observed when other bacterial species are grown in the presence of high concentrations (50 mM to 1.33 M) of glycine [44]. These phenotypes may be due to inhibition of the periplasmic transpeptidation reactions, leading to a lower degree of crosslinkage between glycan strands and thus reducing the integrity of the PG mesh and its resistance to osmotic pressure. Indeed, lower levels of PG crosslinkage were observed when *C. crescentus* and several other bacteria were grown in media supplemented with high concentrations of glycine [23], [44]. Under our conditions of low glycine concentration in the media, the degree of PG crosslinkage is only minimally affected, if at all (Fig. 1C and 2C, Tables 2 and 4), despite the significant accumulation of Penta(Gly) side chains in the PG. The pattern of PG growth (i.e., pre-septal elongation of new PG material around the midcell) also appears unaffected by the change in the PG (and presumably Lipid II precursor) composition (Fig. 3). This apparent plasticity in the behavior of *C. crescentus* may be important for growth in biofilm communities [50], where local concentrations of nutrients, including glycine, may reach substantial levels due to release from dying cells.
